# Rapid dissemination of colistin and carbapenem resistant *Acinetobacter baumannii* in Central Greece: mechanisms of resistance, molecular identification and epidemiological data

**DOI:** 10.1186/s12879-015-1297-x

**Published:** 2015-12-09

**Authors:** O. Oikonomou, S. Sarrou, C. C. Papagiannitsis, S. Georgiadou, K. Mantzarlis, E. Zakynthinos, G. N. Dalekos, E. Petinaki

**Affiliations:** Department of Microbiology, University Hospital of Larissa, Larissa, Greece; Faculty of Medicine and University Hospital in Plzen, Charles University in Prague, Plzen, Czech Republic; Department of Medicine, Medical School, University of Thessaly, Larissa, Greece; Department of Critical Care, Medical School, University of Thessaly, Larissa, Greece; Department of Microbiology, Medical School, University of Thessaly, Larissa, Greece

**Keywords:** *A. baumannii*, Colistin resistance, ST101, Greece

## Abstract

**Background:**

Colistin-resistant/carbapenem-resistant *Acinetobacter baumannii* is a significant challenge for antibiotic treatment and infection control policies. Since 2012, in Central Greece an increase of colistin/pan- resistant *A. baumannii* has occurred, indicating the need for further analysis.

**Methods:**

A total of 86 colistin-resistant/carbapenem-resistant out of 1228 *A. baumannii* clinical isolates, consecutively collected between 2012 and 2014 in a tertiary Greek hospital of Central Greece, as well as one environmental isolate from surveillance cultures were studied. Molecular typing and mechanisms of resistance to colistin and to carbapenems were assessed, whereas, epidemiological and clinical data of the patients were reviewed.

**Results:**

During the study period, the rate of colistin resistance gradually increased and reached 21.1 % in 2014. All colistin-resistant/carbapenem-resistant *A. baumannii* belonged to 3LST ST101 clone that corresponds to the international clonal lineage II. Carbapenem resistance was associated with the presence of *bla*_oxa-23-like_, while resistance to colistin probably correlated with G54E and R109H amino acid substitutions in PmrA and PmrC, respectively.

**Conclusions:**

Epidemiological data of the patients indicated that the first detection of colistin-resistant/carbapenem-resistant ST101 clone in the University Hospital of Larissa (UHL) was associated with a patient who previously had received colistin, while, the movement of the infected patients into the hospital probably resulted to its spread.

## Background

*Acinetobacter baumannii* isolates are recognized as major nosocomial pathogens; their ability to survive in dry conditions and their resistance to disinfectants allow these microorganisms to survive in the hospital environment [1, 2]. Carbapenems, which have been used to treat infections caused by *A. baumannii*, are gradually being inactivated by the emergence of resistant strains [3]. Various mechanisms involved in carbapenem-resistance of *A. baumannii* include production of carbapenemases, modification of antibiotic targets, overexpression of efflux pumps and loss of porins [4]. Carbapenem-resistant strains that are also simultaneously resistant to various, non-beta-lactams antimicrobial agents pose important therapeutic problem, due to the limited number of efficacious drugs [5]. Colistin and tigecycline are often the only treatment options for multiresistant *A. baumannii* infections but resistance to both agents has recently been described, with colistin resistance scattered worldwide [6–13].

Colistin is rapidly bactericidal for Gram-negative bacteria, interacting with the lipid A components of lipopolysaccharide (LPS), resulting in the disorganization of the outer membrane of bacteria [14]. However, the wide usage of colistin, against carbapenem-resistant multiresistant *A. baumannii* led to the development of resistance [15]. In *A. baumannii*, colistin-resistance is mediated by complete loss of LPS production via mutations within the genes (*lpxA*, *lpxC* and *lpxD*) of the lipid A biosynthesis pathway or by modification of lipid A components of LPS via mutations in the *pmrA* and *pmrB* genes of the two-component regulatory system and *pmrC* that codes a lipid A phosphoethanolamine transferase [16–20].

In the University Hospital of Larissa (UHL), that is the main tertiary hospital of Thessaly (Central Greece) serving a region of 1,200,000 habitants, enhanced systemic surveillance resulted to a decrease of carbapenem-resistant *A. baumannii* between 2012 and 2014 (2.6 cases in 2012 vs 2.1 cases in 2013 and 1.9 cases in 2014 respectively, per 1,000 patient-days*).* However, despite this encouraging record, increased rate of colistin-resistance among *A. baumannii* was observed. Aim of this study was to determine the molecular characteristics of the isolates (mechanisms of resistance and molecular typing) and to describe the epidemiological traits.

## Methods

### Clinical isolates, identification and susceptibility testing

From January 2012 to December 2014, a total of 1228 consecutive *A. baumannii* isolates (494 in 2012, 407 in 2013 and 327 in 2014) were recovered from clinical samples taken as part of standard care of an equal number of individual patients admitted to the University Hospital of Larissa, Thessaly (Central Greece). Only the first isolate from each patient was included in this study. In addition, ten environmental isolates, recovered during 2014 from 300 environmental cultures (100 from sink, 50 from patient beds, 50 from cupboards and 100 from handle of door) from various wards of UHL, were also included. Cotton-tipped swabs were moistened with sterile saline and surfaces vigorously scrubbed. Potential fomites included ventilator control panel and sensor, bed rails, supply cabinets, computer keyboards and mice, curtains, air vents, door handles, supply carts and mattresses.

### Identification and susceptibility testing

Identification and antimicrobial susceptibility testing of bacterial isolates was performed by the VITEK-2 system (bioMérieux, Marcy l’Étoile, France). Determination of MICs to colistin and fosfomycin were assessed by the Etest method (bioMérieux, Marcy l’Étoile, France), while, the interpretation was in accordance with the CLSI breakpoints [21]. For isolates with MICs to colistin > = 4 mg/L confirmation was done by broth microdilution method according to CLSI guidelines [22]. *Escherichia coli* ATCC 25922 and *Pseudomonas aeruginosa* ATCC 27853 were used as control strains [23].

### Molecular assays

All *A. baumannii,* clinical and environmental, that were classified as colistin-resistant using CLSI criteria (MICs > =4 mg/L), were further analyzed for molecular typing and the underlying mechanisms of resistance to colistin and to β-lactams.

- Molecular typing

Molecular typing was done using primers for amplifying and sequencing *ompA*, *csuE* and *bla*_OXA-51-like_ gene fragments as described previously [24].

b. Mechanisms for colistin and carbapenem resistance

The strains were screened for the presence of mutations in the *lpx*A, *lpx*C, *lpx*D and *pmr*CAB genes, which have been associated with the emergence of colistin resistance. After DNA extraction by using the Quick-gDNA™ MiniPrep kit (ZYMO RESEARCH Corp., USA), the genes were amplified by polymerase chain reaction (PCR) as described previously [16, 19], and the PCR amplicons were sequenced on both DNA strands using an ABI3730 DNA sequencer (Applied Biosystems, Warrington, United Kingdom). Nucleotide sequences of *lpx*A, *lpx*C, *lpx*D and *pmr*CAB genes from colistin-resistant isolates were compared with the respective sequences obtained from three colistin-susceptible (MICs to colistin 0.25 mg/ml,), belonging to the same clone as the resistant strains, and with that of the reference strain ATCC17978 (GenBank Accession Number CP000521). The detection of *bla*_IMP,_*bla*_VIM,_*bla*_GIM,,_*bla*_SIM,_*bla*_KPC,_*bla*_NDM-1_ and *bla*_oxa-48,_ as well as *bla*_oxa-23-like_*bla*_oxa-23-like_, *bla*_oxa-24-like_, *bla*_oxa-51-like_, *bla*_oxa-58-like_, was done by PCR as described elsewhere [25].

### Demographic data of patients

Demographic data (age, sex etc.) and clinical data of patients diagnosed with colistin- resistant *A. baumannii* infection were reviewed regarding their current and previous hospitalizations, previous antibiotic therapy, sites of infection or colonization, comorbidities, outcome etc.

### Ethics

Before obtaining the clinical information, all patients sign the inform consent, while, approval was received by the Ethics Committee of the UHL that is represented by the Infection Control Committee (number of permission 1334).

## Results

During the study period, 1116 carbapenem-resistant *A. baumannii* (CR *A. baumannii*) were isolated. The rate of carbapenem resistance was 93 % in 2012, 88 % in 2013 and 91 % in 2014, respectively, while, all carbapenem-resistantisolates had MICs to imipenem and meropenem >256 mg/L. On the other hand, the rate of colistin resistance was 1 % in 2012, 2.9 % in 2013 and 21.1 % in 2014, while, an increase in MIC_mean value_, MIC_50_ and MIC_90_ was observed from year to year. In more details, the MIC_mean value,_ MIC50 and MIC90 were 0.76, 0.48 and 0.49 mg/L in 2012, 1.44, 0.49 and 0.62 mg/L in 2013 and 6.4, 0.65 and 3.3 mg/L in 2014, respectively.

Totally, eighty six *A.baumannii* clinical isolates were identified as colistin- and carbapenem-resistant, with the MICs of colistin ranging from 16 to 64 mg/L; they were recovered from clinically significant samples (blood, bronchial secretions etc.) from different patients of the UHL and all were associated with infection according to criteria [26–28]. Five and 12 of them were collected in 2012 and in 2013, respectively, while the remaining 69 *A. baumannii* were eventually collected during 2014. All were pan-resistant expressing simultaneously resistance to fluoroquinolones and aminoglycosides, whereas, 13 and 40 % of them were susceptible to tigecycline and fosfomycin, respectively. No decrease in resistance levels of other antimicrobial agents was observed. On the other hand, the environmental *A. baumannii* were carbapenem-resistant, but only one was resistant to colistin.

Molecular characterization of the isolates (86 clinical and one environmental) revealed that all belonged to 3LST ST101, that corresponds to the international clonal lineage II, and carried the *bla*_OXA-23_ gene being responsible for high level resistance to carbapenems (MIC >256 mg/L). To investigate the mechanisms of the observed high rate of resistance to colistin, all colistin-resistant isolates were screened for the presence of mutations in the *lpxACD* and *pmrCAB* genes. Sequencing of *lpx* genes revealed the presence of four amino acid substitutions (LpxA: Y131H; LpxC:C120R + N287D; LpxD:E117K) in all colistin-resistant and colistin-susceptible isolates, compared to the sequences of the *A. baumannii* ATCC 17978. On the other hand, sequencing of the *pmrCAB* genes detected two different patterns of amino acid substitutions, that were not detected in the ATCC 17978 strain, (i) PmrB:A138T + A226V + A444V; PmrC:F150L + I212V + R332K + A354S + K515T (pattern A; GenBank accession number KR024312) among colistin-susceptible ST101 *A. baumannii* and (ii) PmrA:G54E; PmrB:A138T + A226V + A444V; PmrC:R109H + F150L + I212V + R332K + A354S + K515T (pattern B; GenBank accession number KR024313) among colistin resistant ST101 *A. baumannii*. This finding apparently underlines the involvement of PmrA: G54E and PmrC: R109H substitutions in the development of colistin resistance in *A. baumannii* isolates of ST101. None of the substitutions, found in *lpxACD* and *pmrCAB* genes, have been previously reported.

According to the demographic data, the index case was a 62 year- old man, hospitalized in the Department of Internal Medicine (DIM) for a cellulitis in the abdominal area, and empirically treated with intravenous colistin and daptomycin; the tenth day of treatment, on 7^th^ January 2012, the patient presented fever and the blood culture obtained recovered a colistin/carbapenem resistant *A. baumannii.* Few days later, two other DIM patients were found to be infected by this microorganism, while, on September, a fourth DIM patient, admitted from a Long Term Care Facility (LTCR) of Thessaly, was diagnosed with pyelonephritis due to this strain. Unfortunately, the patient deteriorated and was transferred to the Intensive Care Unit (ICU) of UHL; 10 days later, an ICU patient, who had never been treated with colistin, developed VAP (ventilator associated pneumonia) due to colistin/carbapenem resistant *A. baumannii*. After the first detection of this pan-resistant microorganism, the Infection Control Committee of UHL applied enhanced hygiene and environmental cleaning measures. From October 2012 to March 2013, no colistin-resistant *A. baumannii* were detected in our hospital. However, between April and June 2013, two small outbreaks took place in both the ICU and the DIM, including five and seven patients respectively. Nevertheless, despite the strict prevention measures, during 2014 several colistin/carbapenem resistant *A. baumannii*., have been isolated in various wards of UHL. Fosfomycin and or tigecycline were mainly used for the treatment of these patients. Clinical information and the outcome of the patients are described in Table 1.

**Table 1:** Clinical characteristics of the patients

*Sex* (Male)		48 %
*Age* (Years)		67 (59,74)
*Days after admit to culture*		10 (6, 17)
*Prior use of colistin within 6 months*		23 %
*Hospitalization within 6 months*		31 %
*Prior use of colistin during hospitalization*		41 %
*ICU stay*		62 %
*Presence of medical devices*		68 %
*Comorbidities*	Chronic Heart disease	31 %
	Chronic Lung disease	10 %
	Diabetes Mellitus	10 %
	Chronic Renal failure	10 %
	Neurological disease	17 %
	Chronic Liver Disease	6 %
	Malignancy	3 %
*Source of infection*	Blood Stream Infection	50 %
	Pneumonia	42 %
	Ventriculitis	6 %
	other	2 0025

Data are presented as median (25 %, 75 % quartiles) or *n* (%)

## Discussion

*Acinetobacter baumannii* is a particularly challenging pathogen because it is associated with a high degree of resistance, and it is difficult to eliminate its environmental reservoir in healthcare settings [29]. Carbapenems are considered first-line agents for the treatment of *A. baumannii* infections, and therefore the rise of infections due to carbapenem-resistant strains is of particular concern [30]. Additionally, carbapenem-resistant *A. baumannii* isolates are often susceptible to only 1 or 2 agents, making them extensively drug-resistant (XDR) pathogens by definition [30]. The incidence of XDR *A. baumannii* infections is continually rising [31–33]. For severe XDR *A. baumannii* infections, polymyxins are frequently used, and are considered by most to be the drugs of choice [34]. Today, colistin-resistant/carbapenem-resistant pan-resistant *A. baumannii*, spread and cause nosocomial outbreaks [35–37].

In UHL such as in all Greek hospitals, the rate of carbapenem-resistant *Klebsiella pneumoniae*, *Pseudomonas aeruginosa* and *Acinetobacter baumannii* is very high, resulting to a wide usage of colistin [38]. Between 2012 and 2014, despite the strict prevention measures in our hospital, the rate of colistin-resistant/pan-resistant *A. baumannii* dramatically increased. All these isolates belonged to 3LST ST101, a clone that predominates for long time in UHL [39]. We note that, since 2011, all colistin-susceptible carbapenem-resistant *A. baumannii* belong to clone ST101 as those exhibiting resistance. We suppose that under the selective pressure of colistin this clone became resistant and then, it was spread from patient to patient. Recently, Mavroidi et al., reported two 3LST ST 101 colistin-resistant *A. baumannii* recovered from patients hospitalized in a tertiary Greek hospital in Athens [40].

In our study, the prevalence of colistin-resistant *A. baumannii* in the environmental cultures was low. Numerous studies have documented the presence of *Acinetobacter* spp in the hospital environment, but rates of positive cultures may vary widely, depending on the epidemiological setting and on the places of collection [1, 41]. Our environmental *A. baumannii* strains originated mainly from the patient beds and the bedside cupboards indicating that these places present risk for nosocomial outbreaks. The ability of this microorganism to form biofilms blocks its eradication from the surfaces by the cleaning and promotes its persistence in the hospital setting [42].

Previous studies have shown that resistance to colistin is associated with mutations in *pmrB* gene [18, 19, 35, 37]. Interestingly, in our strains, colistin resistance was correlated with mutations in *pmrA* and *pmrC*. However, a further study is necessary to prove the involvement of the G54E (PmrA) and R109H (PmrC) amino acid substitutions in the development of colistin resistance in *A. baumannii*, and to characterize the impact of the latter substitutions in the modification of lipid A components. Of note was that, acquisition of colistin resistance did not alter resistance to other antibiotics, which is in agreement with previous studies demonstrating that, in contrast to Lpx alterations, mutations in *pmrCAB* do not result in increased susceptibility to other antimicrobials [16, 35].

The UHL is the main tertiary care unit of Central Greece and admits patients coming from different parts of the area; therefore, there is a great risk of spreading this pan-resistant clone in the entire region, concerning that this clone consist part of the microbiota of the hospital. The first four months of 2015, the rate of colistin/carbapenem resistant *A. baumannii* continued rising dramatically and reached to 52 % (data not shown). In order to limit this dissemination, all patients before their discharge are tested if they are colonized with colistin-resistant *A. baumannii*. In addition, unnecessary transfer of patients from the surrounding hospitals to UHL stopped. Until now, only few colistin-resistant strains have been isolated from the local hospitals and LTCFs of Central Greece.

## Conclusions

In the present study, from 2012 till 2014, a significant increase of colistin-resistant/carbapenem-resistant *A. baumannii* in a tertiary care hospital in central Greece was observed. All isolates belonged to 3LST ST101 clone and their resistance to carbapenems and to colistin was associated with the production of OXA-23-like carbapenem-hydrolyzing class D β-lactamase and with mutations in *pmrA* and *pmrC* genes*,* respectively. Epidemiological data of the patients indicated that the first detection of colistin-resistant/carbapenem-resistant ST101 clone in UHL was associated with a patient who had previously received colistin, while, the movement of the infected patients into the hospital probably resulted to its spread.

## Abbreviations

DIM: Department of Internal Medicine
ICU: Intensive Care Unit
LST: locus sequence typing
LTCF: Long Term Care Facility
ST: sequence type
UHL: University Hospital of Larissa
